# Decoupling musculoskeletal damage from antiviral control: the NLRP3 inflammasome in arthritogenic alphavirus infection

**DOI:** 10.3389/fcimb.2026.1880650

**Published:** 2026-07-09

**Authors:** Fahu Yuan, Jiangyuan Chen

**Affiliations:** 1 School of Medicine, Jianghan University, Wuhan, China; 2 State-owned Assets and Laboratory Management Office, Jianghan University, Wuhan, China

**Keywords:** arthritogenic alphavirus, Chikungunya virus, Mayaro virus, NLRP3 inflammasome, Ross River virus, therapeutic decoupling, viral arthritis

## Abstract

Mosquito-borne arthritogenic alphaviruses, including chikungunya virus (CHIKV), Ross River virus (RRV), and Mayaro virus (MAYV), cause acute febrile polyarthralgia and can leave a substantial proportion of patients with persistent or relapsing musculoskeletal disease. In many cohorts, chronic arthritis continues after systemic viremia has declined, suggesting that late tissue pathology is sustained largely by host inflammatory programs rather than by ongoing productive viral replication. This Mini Review examines the NLRP3 inflammasome as a pathogenic node linking viral danger sensing to chronic joint and bone damage, and considers whether this pathway can be targeted to reduce tissue injury while preserving antiviral control. Across CHIKV, RRV, and MAYV models, pattern-recognition receptor signaling provides NF-κB-dependent priming of NLRP3, pro-IL-1β, and pro-IL-18, whereas K^+^ efflux, mitochondrial stress, reactive oxygen species, and lysosomal disruption promote NLRP3–ASC–caspase-1 assembly in musculoskeletal niches. The resulting IL-1β and IL-18 release and, in selected contexts, pyroptosis-associated amplification propagate inflammatory signaling across monocytes/macrophages, fibroblast-like synoviocytes, osteoblasts, and osteoclast-lineage cells, thereby promoting synovitis, myositis, osteoclastogenesis, and bone loss. Pharmacological and genetic studies indicate partial phenotypic separation between tissue damage and viral control, as NLRP3 inhibition or IL-1 receptor blockade can attenuate musculoskeletal pathology with limited effects on viral burden in available preclinical models. We highlight three priorities for translation: defining post-acute therapeutic windows, stratifying patients using inflammasome and bone-turnover biomarkers, and incorporating bone-centered endpoints such as micro-computed tomography and osteoclast histomorphometry.

## 1 Introduction

Mosquito-borne arthritogenic alphaviruses, principally chikungunya virus (CHIKV), Ross River virus (RRV), and Mayaro virus (MAYV), continue to pose an expanding threat to global health (de Souza et al., 2025; Suhrbier et al., 2012). These positive-sense single-stranded RNA viruses of the *Togaviridae* family share common mosquito vectors, particularly *Aedes aegypti* and *Aedes albopictus*, and show a marked tropism for the musculoskeletal system. After large outbreaks in the Americas between 2014 and 2016, CHIKV has maintained autochthonous transmission across multiple continents, accounting for millions of infections. RRV remains the most prevalent arboviral disease in Australia and the Pacific, whereas MAYV is increasingly recognized beyond its traditional endemic settings in South America, with spillover events linked to the interface between sylvatic and urban transmission cycles (de Souza et al., 2025).

The clinical burden of arthritogenic alphavirus infection is not limited to the acute febrile episode. Patients typically present with high fever, maculopapular rash, and severe polyarthralgia, but a substantial proportion—estimated at 30–60%, depending on the cohort and viral strain—develop persistent joint pain, morning stiffness, and functional impairment that may last for months or years (Suhrbier et al., 2012). This chronic syndrome often resembles seronegative rheumatoid arthritis and can impose substantial disability and economic costs in endemic regions. Importantly, musculoskeletal symptoms may persist long after peripheral viremia has declined or become undetectable, indicating that late-phase pathology is sustained largely by host inflammatory programs rather than by ongoing productive viral replication. This temporal dissociation raises a central therapeutic question: can tissue-damaging inflammation be selectively attenuated without compromising antiviral immunity?

The NLRP3 inflammasome provides a useful framework for understanding this decoupling hypothesis (Swanson et al., 2019). By integrating viral pathogen-associated molecular patterns (PAMPs) with damage-associated molecular patterns (DAMPs) generated during cellular stress, NLRP3 links innate viral sensing to the maturation and release of interleukin-1β (IL-1β) and IL-18. Within the joint microenvironment, this axis is distributed across several interacting cellular niches. Inflammatory monocytes and macrophages are major sources of inflammasome-derived cytokines; fibroblast-like synoviocytes (FLS) sustain chemokine production and matrix remodeling; endothelial cells regulate leukocyte recruitment; and osteoclast-lineage cells translate inflammatory cues into measurable bone loss. Recent spatial transcriptomic analyses of CHIKV-infected murine joints have identified joint-associated macrophage populations that retain viral RNA signals and maintain elevated *Nlrp3* and *Il1b* expression into the chronic phase, providing a cellular basis for sustained inflammasome priming after systemic viral clearance (Zarrella et al., 2026). On this basis, this Mini Review examines cross-viral evidence supporting the concept that NLRP3-driven musculoskeletal injury can, at least in part, be separated from alphavirus control. We focus on the mechanistic transition from antiviral defense to chronic immunopathology and discuss how this distinction may guide phase-specific inflammasome-targeted strategies for alphavirus-associated arthritis.

## 2 The NLRP3 inflammasome as a mechanistic hub in musculoskeletal disease

### 2.1 Priming signals and the role of joint-associated macrophages

NLRP3 inflammasome activation is commonly framed as a two-signal process that limits inappropriate inflammatory output under resting conditions. Signal 1, or priming, is initiated by pattern-recognition receptors (PRRs), including endosomal TLR7/8 and cytosolic RIG-I-like receptors, which detect viral single-stranded RNA and replication intermediates. These pathways converge on NF-κB signaling and induce transcription of NLRP3, pro-IL-1β, and pro-IL-18 (Kelley et al., 2019). In alphavirus infection, this priming step is not necessarily confined to the viremic phase. Recent single-cell and spatial analyses of CHIKV-infected joints have identified joint-associated macrophages that retain viral RNA signals weeks after systemic clearance. Such persisting viral signals may maintain low-level PRR–NF-κB activity and keep synovial macrophages poised for inflammasome activation, even in the absence of sustained high-titer productive infection (Zarrella et al., 2026). This localized macrophage reservoir provides a plausible cellular basis for prolonged, tissue-damaging inflammatory responses in chronic alphavirus arthritis.

### 2.2 Activation cues in the alphavirus-infected joint

Signal 2 promotes conformational rearrangement and oligomerization of primed NLRP3, recruitment of apoptosis-associated speck-like protein containing a CARD (ASC), and assembly of the inflammasome complex, culminating in autocatalytic cleavage of pro-caspase-1. In alphavirus-infected cells, activation is likely driven by convergent disturbances in cellular homeostasis. Potassium (K^+^) efflux represents a central cue. Alphavirus 6K protein and its transframe variant TF can display viroporin-like activity, altering membrane permeability and potentially contributing to intracellular potassium depletion, a well-established trigger of NLRP3 activation (Melton et al., 2002). Mitochondrial stress provides a second activation route: viral replication and inflammatory stress can promote mitochondrial dysfunction, reactive oxygen species (ROS) generation, and release of mitochondrial DNA (mtDNA) into the cytosol. In parallel, uptake of viral debris and necrotic material in inflamed joint tissues may overload lysosomes, causing lysosomal membrane permeabilization and cytosolic cathepsin release. Together, these convergent stress signals lower the threshold for NLRP3 inflammasome assembly in infected musculoskeletal niches (Lidbury et al., 2008).

### 2.3 Tissue niches, effector modules, and bone loss

Once activated, caspase-1 converts pro-IL-1β and pro-IL-18 into mature cytokines that amplify local inflammation. Among these outputs, IL-1β is especially relevant to musculoskeletal pathology because it promotes osteoclast differentiation, bone resorption, and inflammatory crosstalk with stromal cells. Active caspase-1 may also cleave gasdermin D (GSDMD), releasing an N-terminal pore-forming fragment that drives pyroptosis and the secondary release of intracellular danger signals (Swanson et al., 2019). These inflammasome outputs do not act in isolation. Within infected or inflamed musculoskeletal tissues, tissue-resident macrophages and recruited mononuclear phagocytes function as a major amplification module, producing mature IL-1β/IL-18 and maintaining a low-grade inflammatory state that can persist beyond the acute viremic phase (Haist et al., 2017).

In parallel, inflammasome-derived cytokines engage a structural damage module within the synovial–bone interface. FLS are highly responsive to paracrine IL-1β and can acquire an inflammatory remodeling phenotype, marked by increased production of matrix metalloproteinases (MMPs), chemokines, and receptor activator of nuclear factor-κB ligand (RANKL). These responses support synovial inflammation, extracellular matrix breakdown, and cartilage damage (Chen et al., 2014). At the same time, IL-1β influences osteoclast precursors and osteoblast-lineage cells, shifting the RANKL/osteoprotegerin (OPG) axis toward osteoclast differentiation and bone resorption. This synovial–osteoclast coupling provides a plausible explanation for why bone loss emerges as one of the most reproducible and measurable structural endpoints in experimental arthritogenic alphavirus disease (Wolf et al., 2019).

**Figure 1:**
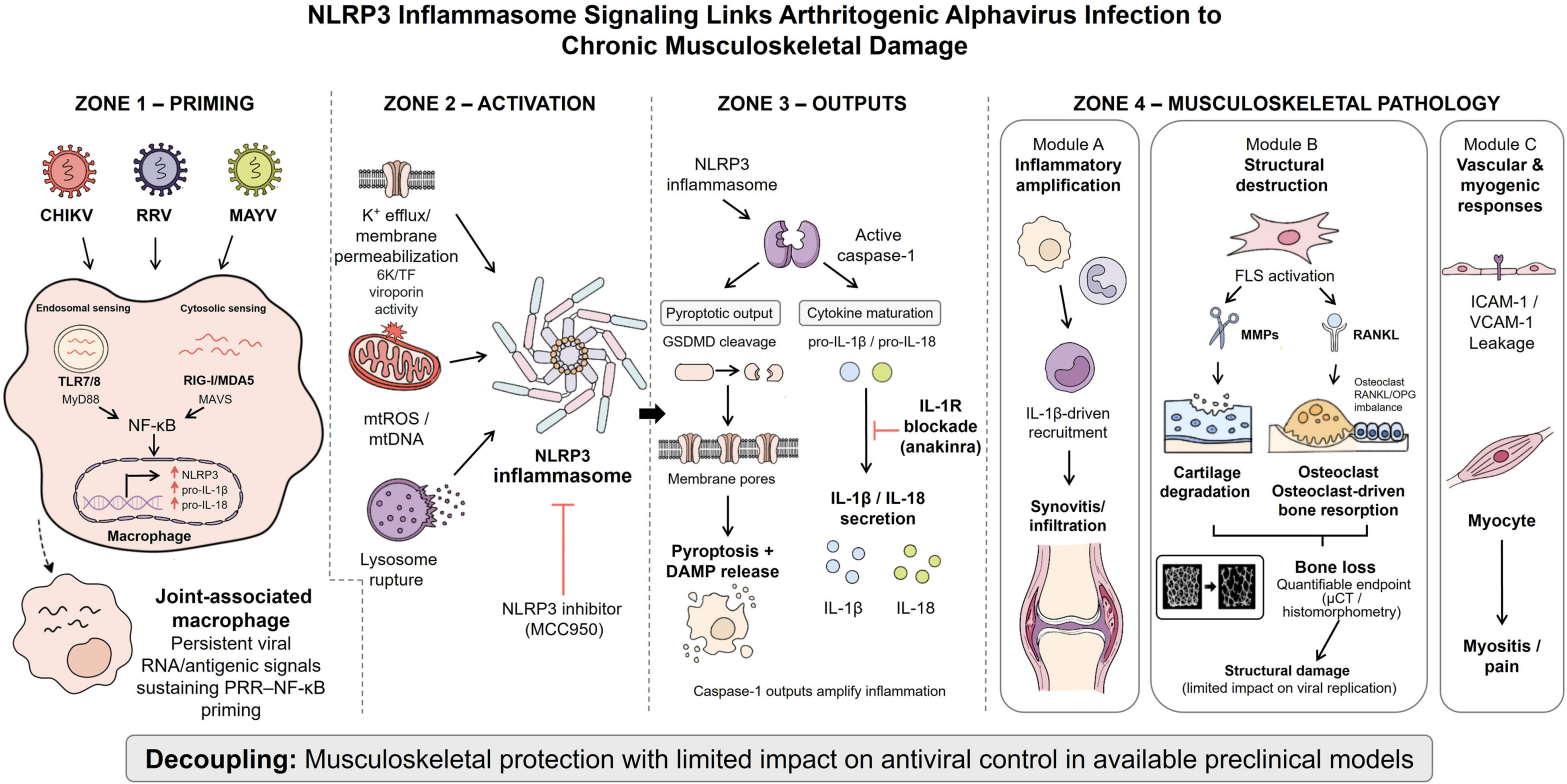
The NLRP3 inflammasome as a mechanistic bridge linking alphavirus infection to chronic musculoskeletal damage. Zone 1 (Priming): Viral RNA signals retained in joint-associated macrophages activate PRRs, driving NF-κB-dependent transcription of NLRP3, pro-IL-1β, and pro-IL-18. Zone 2 (Activation): Convergent cellular stress cues, including K^+^ efflux associated with 6K/TF viroporin activity, mitochondrial ROS, and lysosomal damage, promote assembly of the NLRP3–ASC–caspase-1 complex. Zone 3 (Outputs): Active caspase-1 processes pro-IL-1β and pro-IL-18 and cleaves GSDMD, promoting cytokine release, pyroptosis, and secondary DAMP release. Zone 4 (Musculoskeletal pathology): These outputs promote local inflammatory amplification, FLS activation, cartilage degradation, osteoclast-driven bone resorption, and vascular and myogenic responses. Red nodes indicate potential sites of therapeutic decoupling, at which MCC950 or anakinra attenuates tissue damage with limited effects on viral burden in available preclinical models.

Taken together, these mechanisms support a tissue-specific inflammasome circuit through which alphavirus sensing can be translated into persistent musculoskeletal pathology. Viral RNA and replication-associated danger signals provide priming through PRR–NF-κB pathways, whereas K^+^ efflux, mitochondrial stress, ROS, and lysosomal disruption converge to promote NLRP3 inflammasome assembly. Activated NLRP3–ASC–caspase-1 signaling drives maturation of IL-1β and IL-18 and, in selected cellular contexts, GSDMD-dependent pyroptosis with secondary DAMP release. These outputs are propagated across macrophages, FLS, osteoblast-lineage cells, and osteoclast-lineage cells, linking innate antiviral sensing to synovitis, pain, osteoclastogenesis, and structural bone loss. This mechanistic framework is summarized in **Figure 1**.

## 3 Comparison with inflammasome activation in other viral infections

A comparison with other viral infections helps define what is distinctive about the translational window in alphavirus arthritis. NLRP3 activation is not unique to arthritogenic alphaviruses; respiratory viruses such as influenza A virus and SARS-CoV-2, as well as flaviviruses such as dengue virus and Zika virus, can engage similar stress pathways, including K^+^ efflux, mitochondrial dysfunction, and ROS generation (Zheng et al., 2020; Wang et al., 2018; Zhao and Zhao, 2020). In some infections, viral proteins may also interact more directly with inflammasome components. For example, Zika virus NS5 has been reported to interact with NLRP3 and promote inflammasome assembly, whereas dengue virus non-structural proteins can enhance NLRP3 activation (He et al., 2018; Shrivastava et al., 2020).

However, the pathological consequences of NLRP3 activation differ substantially according to viral tropism and tissue compartmentalization. In severe respiratory viral infections, such as influenza or COVID-19, inflammasome activation in airway epithelial cells and alveolar macrophages can contribute to pulmonary inflammation, epithelial injury, acute respiratory distress syndrome (ARDS) and systemic inflammatory complications (Mangalmurti and Hunter, 2020; Savidis et al., 2016). In this setting, the therapeutic margin for inflammasome inhibition may be narrow, because inflammation, tissue injury, and viral control often overlap temporally during the acute phase. Some respiratory viruses also encode proteins that modulate inflammasome activity, including influenza PB1-F2, underscoring the evolutionary pressure to tune inflammasome signaling during early host–virus interactions (Pinar et al., 2017).

By contrast, arthritogenic alphaviruses display a marked tropism for musculoskeletal tissues, including fibroblasts, myocytes, and osteoblast-lineage cells. This tropism concentrates much of the clinically relevant pathology within synovial, muscular, and skeletal compartments, where NLRP3–IL-1β signaling can sustain local inflammation and bone remodeling imbalance. The translational significance of this compartmentalization lies in its timing: chronic joint pathology frequently persists after systemic viremia has declined, providing a post-acute interval in which tissue-protective therapy may be pursued with less risk of interfering with early antiviral control. Selective inhibition of NLRP3 or IL-1 signaling may therefore offer a way to limit synovitis, myositis, and bone loss without broad immunosuppression. In this respect, alphavirus arthritis represents a useful disease context for testing therapeutic decoupling of inflammasome-driven tissue injury from viral clearance.

## 4 Cross-viral evidence for a separable pathogenic axis

### 4.1 Human and clinical evidence

Patient-derived data provide the first layer of biological relevance. In CHIKV infection, acute and subacute disease is accompanied by an innate inflammatory signature that includes elevated IL-1β, IL-6, and IL-8, with cytokine levels linked to symptom severity and prolonged arthralgia in some cohorts (Chow et al., 2011). These observations suggest that host inflammatory programs contribute to disease persistence beyond the initial viremic phase. In line with this interpretation, analyses of patient peripheral blood mononuclear cells (PBMCs) and experimental CHIKV infection have associated disease inflammation with activation of the NLRP3 pathway, including increased NLRP3 expression and caspase-1 activity (Chen et al., 2017).

Similarly, acute-phase sera from MAYV-infected patients show a detectable inflammasome-associated signature. Compared with convalescent patients or healthy controls, acute MAYV cases exhibit increased levels of active caspase-1 p20, together with mature IL-1β and IL-18, providing measurable evidence of inflammasome activation in this emerging alphavirus infection (de Castro-Jorge et al., 2019). Clinical observations further suggest that long-term arthralgia after MAYV infection is associated with sustained pro-inflammatory cytokine responses, although the relationship between inflammatory persistence, viral burden, and tissue-specific pathology remains incompletely defined (Tappe et al., 2016; Santiago et al., 2015). Taken together, these human data support biological plausibility but remain largely observational. Interventional studies in relevant animal models are therefore needed to test whether inflammasome-driven tissue damage can be separated from antiviral control.

### 4.2 Animal models and phenotypic decoupling in CHIKV

Pharmacological and genetic intervention studies provide the strongest causal support for the decoupling concept. In C57BL/6 murine footpad infection models, CHIKV induces biphasic swelling, myositis, synovial inflammation, and osteoclast-associated bone loss in the tarsal joints. Using MCC950, a selective small-molecule inhibitor of NLRP3, Chen et al. showed that inflammasome inhibition reduced footpad swelling, synovial inflammation, skeletal muscle pathology, and osteoclast-mediated bone loss. Micro-computed tomography (μCT) analysis demonstrated partial preservation of bone volume and trabecular thickness, indicating that NLRP3 activity contributes not only to inflammatory swelling but also to structural skeletal damage (Chen et al., 2017). Notably, these protective effects were observed without a major increase in viral titers in blood, liver, or joint tissues. These findings provide a key proof of concept for therapeutic decoupling: in this model, NLRP3-driven tissue injury could be reduced while antiviral control was largely preserved.

### 4.3 Phenotypic separation in RRV and MAYV

Downstream IL-1 receptor blockade in RRV infection provides complementary evidence for therapeutic decoupling. In mouse models, RRV induces polyarthritis, myositis, and marked bone loss. Using anakinra, a recombinant IL-1 receptor antagonist, Wolf et al. showed that IL-1 blockade had a stronger effect on skeletal pathology than on aggregate clinical scores. Although improvements in overall disease scores were modest, anakinra preserved bone mineral density, reduced trabecular bone loss, and lowered the number of tartrate-resistant acid phosphatase (TRAP)-positive multinucleated osteoclasts in the epiphysis (Wolf et al., 2019). These structural benefits occurred without a major alteration in systemic viral clearance (Wolf et al., 2019). The study therefore highlights a clinically important form of phenotypic separation: IL-1 signaling appears to be a major driver of osteoclastogenesis and bone resorption, whereas viral control is relatively preserved in this model.

MAYV models provide additional, although less extensive, support for this principle. In experimental MAYV infection, *Nlrp3* deficiency reduced footpad swelling, inflammatory cell infiltration, and mechanical hypernociception, supporting a role for NLRP3 in local inflammatory pathology and pain-like responses (de Castro-Jorge et al., 2019). These effects were not accompanied by increased viral loads in target tissues compared with wild-type controls. Thus, while CHIKV and RRV currently provide the strongest evidence linking inflammasome signaling to osteoclastogenesis and bone loss, MAYV broadens the cross-viral relevance of the decoupling framework. Overall, the available evidence suggests that the NLRP3–IL-1β axis functions more consistently as a local amplifier of musculoskeletal injury than as an indispensable determinant of antiviral control.

## 5 Translational priorities

### 5.1 Phase-specific therapeutic windows

Inflammasome function during viral infection is strongly shaped by disease phase. During acute viremia, inflammasome activation may contribute to early antiviral defense, immune-cell recruitment, and the initiation of adaptive immunity. Broad inhibition at this stage could therefore carry a risk of interfering with viral control. By contrast, persistent inflammasome activity during the post-acute phase may increasingly act as a driver of tissue injury rather than as a protective antiviral response. This creates a potential therapeutic window after peak viremia, when systemic viral burden has declined but macrophage priming, IL-1 signaling, and osteoclast activation may remain active within affected joints. The precise timing of this window is likely to vary by virus, host background, and disease severity, but it is most plausibly located in the early post-acute period rather than during the initial viremic phase. In this setting, phase-specific targeting of the NLRP3–IL-1β axis may offer a more favorable balance between limiting musculoskeletal damage and preserving antiviral immunity. Defining the appropriate treatment window is only one part of the translational challenge; the clinical feasibility of inflammasome-targeted therapy also depends on the safety, pharmacokinetic properties, and stage of clinical development of the available agents.

### 5.2 From preclinical proof-of-concept to clinically viable intervention

Direct NLRP3 inhibition provides important mechanistic proof of concept, but translation from experimental models to human therapy remains challenging. MCC950 demonstrated that inflammasome-driven musculoskeletal injury could be reduced in alphavirus models without a major increase in viral burden. However, its clinical development was discontinued after elevations in liver enzymes were reported, highlighting the need to distinguish compound-specific efficacy from an acceptable therapeutic safety margin in humans (Mullard, 2019). Whether this toxicity reflects the chemical properties of MCC950 or a broader limitation of sustained NLRP3 inhibition remains unresolved.

This experience also illustrates the difference between experimental NLRP3 blockade and clinically established downstream IL-1 inhibition. Anakinra has well-characterized dosing, pharmacokinetics, and safety information from its approved use in inflammatory diseases, making it a more immediately testable option for post-acute alphavirus arthritis. Nevertheless, its known risks, including infection and injection-site reactions, and the absence of efficacy data in human alphavirus disease still require careful consideration. Dapansutrile has shown short-term tolerability and anti-inflammatory activity in early human studies, but its optimal dose, treatment duration, tissue penetration, and effects on antiviral immunity remain to be defined in infection-specific settings (Marchetti et al., 2018; Klück et al., 2020). Thus, future clinical development should evaluate not only anti-inflammatory efficacy but also hepatic safety, systemic exposure, treatment timing, and the potential consequences of inhibiting innate immune signaling during or after viral infection.

Even if a clinically suitable agent becomes available, treatment is unlikely to be appropriate for all infected patients, making biomarker-guided patient selection an equally important translational priority.

### 5.3 Biomarker-guided patient stratification

Because only a subset of alphavirus-infected patients progress to chronic arthritis or structural joint damage, patient selection will be central to clinical translation. A practical stratification strategy could combine inflammasome-related markers, such as IL-1β, IL-18, and caspase-1 activity, with bone-turnover markers including C-terminal telopeptide of type I collagen (CTX-I) and procollagen type I N-terminal propeptide (P1NP). Soluble urokinase plasminogen activator receptor (suPAR) and other systemic inflammatory markers may help capture broader inflammatory burden, but their predictive value in alphavirus-associated arthritis remains to be established prospectively (Rasmussen et al., 2021). Integrating these markers could help identify patients with persistent inflammasome activity and evolving skeletal involvement, thereby improving trial enrichment and reducing unnecessary exposure to anti-inflammatory therapy in patients with rapidly resolving disease.

Biomarker performance is also likely to vary across patient populations. Age, sex, comorbid inflammatory or metabolic disease, baseline immune status, prior exposure to alphaviruses or other arboviruses, and the interval between symptom onset and sample collection may all influence circulating cytokines, caspase-1 activity, and bone-turnover markers. Viral strain, disease severity, and previous anti-inflammatory treatment may introduce additional variability. These factors are especially relevant in endemic regions, where repeated arboviral exposure and clinically overlapping infections may complicate interpretation. Candidate biomarkers should therefore be validated in longitudinal, geographically diverse cohorts using standardized sampling windows, well-defined clinical phenotypes, and objective imaging or bone-remodeling outcomes before they are used to guide patient selection in intervention trials.

### 5.4 Standardized bone-centered endpoints

A key lesson from RRV and CHIKV animal models is that symptom-based disease scores and structural bone outcomes may not change in parallel. Future therapeutic studies should therefore give greater weight to bone-centered endpoints when evaluating inflammasome- or IL-1-targeted interventions. These endpoints may include μCT-derived trabecular parameters, such as bone volume fraction (BV/TV), trabecular thickness, and trabecular number, together with histomorphometric quantification of osteoclast numbers. Serum bone-turnover markers, including CTX-I for bone resorption and P1NP for bone formation, may provide complementary non-invasive readouts. Patient-reported pain scales and functional indices remain essential, but structural metrics can capture tissue-protective effects that may be missed by global clinical scores alone. Preventing persistent bone loss and long-term skeletal sequelae should therefore be considered a clinically meaningful goal in future studies of alphavirus-associated arthritis (Wolf et al., 2019; Chen et al., 2017).

## 6 Conclusion and future directions

The NLRP3 inflammasome provides a mechanistic bridge between arthritogenic alphavirus infection and chronic musculoskeletal immunopathology. Across CHIKV, RRV, and MAYV models, available evidence supports a partial separation between viral control and inflammasome-driven tissue injury, most clearly in the post-acute phase when systemic viremia has declined but local IL-1 signaling, macrophage priming, and osteoclast activation may persist. This temporal and anatomical separation distinguishes alphavirus arthritis from viral syndromes in which inflammasome activation is tightly coupled to acute systemic inflammation. MCC950 has established NLRP3 inhibition as a preclinical proof of concept, although its clinical translation has been constrained by safety concerns. Newer NLRP3 inhibitors, including dapansutrile (OLT1177), and approved IL-1 pathway inhibitors such as anakinra offer realistic tools for testing this strategy in clinically relevant settings (Marchetti et al., 2018; Dinarello et al., 2012). Future progress will depend on matching intervention to disease phase, selecting patients with persistent inflammasome activity, and measuring structural outcomes such as bone loss. In this framework, therapeutic decoupling is not simply anti-inflammatory treatment; it is a stage-specific strategy to limit chronic skeletal damage while maintaining antiviral defense.
